# The Selective Detection of Individual Respiratory Droplets in Air

**DOI:** 10.1021/acssensors.5c02057

**Published:** 2025-12-16

**Authors:** Matjaž Malok, Darko Kavšek, Maja Remškar

**Affiliations:** † 61790 Jozef Stefan Institute, 1000 Ljubljana, Slovenia; ‡ Faculty of Mathematics and Physics, University of Ljubljana, 1000 Ljubljana, Slovenia; § Nanotul Ltd., 1000 Ljubljana, Slovenia

**Keywords:** respiratory droplets, airborne transmission, capacitive sensing, indoor air monitoring, infection control

## Abstract

Preventing the spread of airborne diseases in crowded indoor spaces is a global challenge. Infected individuals release virus-laden respiratory droplets (RDs) that can remain suspended in air and infectious for hours. Current monitoring methods cannot distinguish these droplets from airborne particulate matter (PM) in a real time. Here, we present a capacitive sensor that selectively detects and counts the individual droplets in indoor spaces, regardless the presence of PM. The device exploits the dielectric constant (ε) of water (78.2) to differentiate the droplets from solid PM particles (ε < 15). In a nonventilated conference-room study, RDs concentrations (40–330 RDs/L) were found to be correlated with human occupancy, but not with PM_2.5_ levels. The developed technology enables a real-time monitoring of number concentration of RDs, which represent a potential health risk when they carry viral or bacterial infections. The detected increase in RD concentration can serve as a trigger for data-driven ventilation and infection-prevention measures, providing an effective tool for mitigating the spread of respiratory diseases in hospitals, schools and other public spaces.

## Graphical abstract

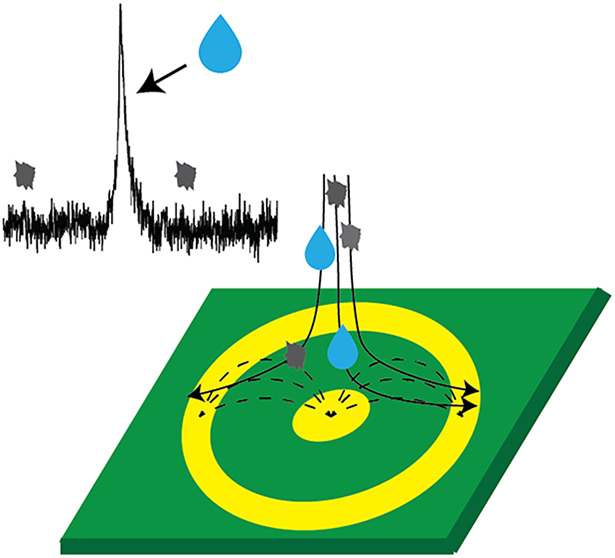

The spread of airborne diseases in crowded indoor environments is a global concern, particularly during pandemics and influenza outbreaks. Viruses are recognized as the most common cause of infectious diseases being transmitted indoors. The primary viral agents responsible for respiratory infections include influenza viruses, rhinoviruses, respiratory syncytial viruses (RSVs), parainfluenza viruses, and coronaviruses, like SARS-CoV-2.^1–3^ Infected individuals release respiratory droplets (RDs) during activities such as breathing, speaking, coughing, sneezing, and other exhalations like yawning, snoring, or shouting.^1^

Larger RDs settle rapidly due to gravity, whereas smaller droplets remain airborne and are transported by the airflows.^4,5^ Although volatile components, including water in RDs, begin to evaporate immediately after exhalation, aerosolized RDs do not dry out completely at normal room conditions.^6^ Exhaled droplets eventually reach an equilibrium state at which their size no longer changes.^7^ These partially dehydrated, aerosolized droplets can remain suspended for prolonged periods, accumulate in indoor environments, and transport viruses that remain infectious for extended durations.^8^

Understanding the spatial distribution and temporal dynamics of RD concentrations is critical for the effective control of airborne-disease transmission in indoor environments. This has been highlighted in multiple theoretical investigations,^9–13^ and supported by experimental studies employing simulated droplets made from atomized 1% NaCl water solutions^14,15^ or atomized *Staphylococcus albus* bacteria.^16^ The detection of airborne SARS-CoV-2 RNA in indoor air is limited to a postcollection laboratory analysis involving bioaerosol sampling followed by a reverse transcription-quantitative polymerase chain reaction (RT-qPCR).^17–20^ These procedures are labor-intensive, require trained personnel, and do not provide in situ or real-time data on RD concentrations.

Direct measurements of RDs have mostly been limited to regions immediately adjacent to the exhalation source.^9,10,21–24^ These studies typically employ complex instrumentation in controlled or clean environments to minimize the background interference. As such, their findings are not directly transferable to real-world indoor settings and are unsuitable for the continuous or occasional monitoring of the droplet concentrations in occupied spaces.

To the best of our knowledge, there have been no prior reports of a dedicated instrument designed specifically for the selective detection of RDs. In this context, selectivity refers to a device’s ability to exclusively detect droplets, despite the presence of solid aerosols in the air. Existing air-quality monitoring devices, such as optical particle counters (OPCs) or condensation particles counters (CPCs), are capable of detecting both solid aerosols and droplets.^25^ However, these systems do not provide for the selective detection of droplets. This limitation is particularly important, as the number concentration of solid aerosols in ambient air is several orders of magnitude higher than that of RDs, and the signals from RDs are masked by the overwhelming presence of solid particulate matter (PM).

In this study, we present a novel method and a device capable of the selective detection of individual droplets in indoor air. The approach is based on a capacitive sensing principle, which was previously employed for the detection of aerosolized PM^26–30^ and simulated RDs with micrometre sizes.^31,32^ The device differentiates the water containing droplets from the solid particles by exploiting the disparity in their dielectric properties. It is capable of continuous, real-time, and selective monitoring of the droplets in indoor air. As such, it holds promise for mitigating the spread of respiratory pathogens in indoor environments.

## Results

### Method and Device for Detecting Respiratory Droplets

The basic principle of RD detection is a change in the capacitance of a sensor when an aerosol particle enters the sensor’s electric field (Figure 1a). The capacitive sensor, manufactured on a 4-layer printed-circuit board (PCB), consists of two coaxial, planar, electrically separated electrodes covered with a few-μm-thick dielectric film having a permeability of 22. One electrode is connected to a virtual ground and the other is biased with a constant voltage. Air is sucked into the device with a flow rate of 1 L/min through a narrow nozzle (0.4 mm in diameter), which accelerates the aerosols to 130 m/s and directs them toward the sensor. When an aerosol particle (either a solid particle or a droplet) enters the sensor’s electric field, it displaces an equal volume of air. Since the aerosol particle has a different dielectric constant than air, this causes a change in the sensor’s capacitance. The change in capacitance (Δ*C*) is then converted by a charge-sensitive amplifier into a voltage pulse (Δ*V*). The impact of a single detected droplet creates a signal (Figure 1b), while the impacts of solid particles are not resolved. This selectivity of droplet detection is based on the fact that solid indoor-air pollutants, such as carbon-based particles, as a major combustion product, have a dielectric constant of less than 15,^33^ while the dielectric constant of water, which is the main component of RDs, is approximately 78.2.^34^ The impact of droplets with significantly different dielectric constants to that of air causes a detectable change in the sensor’s capacitance. On the other hand, when particles or other substances with dielectric constants similar to that of air enter the sensor, the resulting changes in capacitance generate electrical signals that are indistinguishable from electronic noise.

**Figure 1:**
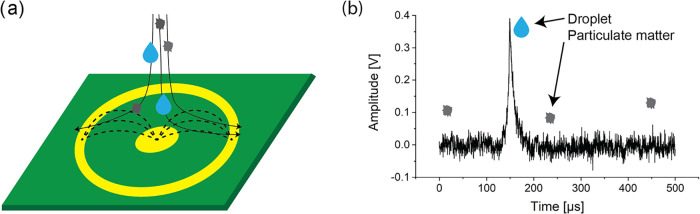
(a) Schematic representation of a device for the selective counting of individual droplets. (b) Detected signal corresponding to the impact of a single droplet.

### Selectivity

The selectivity was experimentally demonstrated by simultaneously measuring aerosols with different dielectric constants using the presented method and a condensation particle counter (WCPC 3785, TSI). The tested particles were water (ε ≈ 78.2) and ethanol droplets (ε ≈ 25),^35^ particles from a smoldering incense stick composed of hydrocarbons (ε = 2.0–6.0, depending on moisture content),^36^ and cigarette smoke (ε = 1.8).^37^ Measurements were carried out under room conditions (23 °C, 40–60% relative humidity (RH)) by either spraying liquids (water or ethanol) or positioning the smoldering incense stick and cigarette near the inlets of both devices. As shown in Figure 2 (bottom row), the WCPC successfully detected all the particle types: water droplets, ethanol droplets, and smoke particles from both the incense stick and the cigarette. The background particle concentration was approximately 1.3 × 10^3^ #/cm^3^. The peak concentrations detected with the WCPC were: 1.65 × 10^5^ #/cm^3^ (water droplets), 4.8 × 10^5^ #/cm^3^ (ethanol droplets), 4.5 × 10^6^ #/cm^3^ (incense smoke), and 1.6 × 10^7^ #/cm^3^ (cigarette smoke). In contrast, the droplet detector (Figure 2, top row) detected only the water and ethanol droplets because of the higher dielectric constants compared to air and carbonaceous smoke particles. The only exception was a minor response to the incense stick, which could be explained by the emission of CaCO_3_ and SiO_2_ particles, identified as the primary emission components.^38^ SiO_2_ particles have a dielectric constant of approximately 3.9,^39^ comparable to that of carbon-based nanoparticles, while CaCO_3_ has a higher dielectric constant ranging from 8.31 to 8.69.^40^ Additionally, the response can also be attributed to the release of water vapor during burning,^41^ which can adsorb onto hydrophilic CaCO_3_ particles,^42^ thereby enhancing their dielectric constant and detection efficiency. Despite this minor response, the measured concentrations were at least 4 orders of magnitude lower than the total particle concentrations recorded by the WCPC. The fact that the droplet detector responded exclusively to water and ethanol droplets, and not to smoke particles from incense or cigarettes, provides evidence of its dielectric-based selectivity.

**Figure 2:**
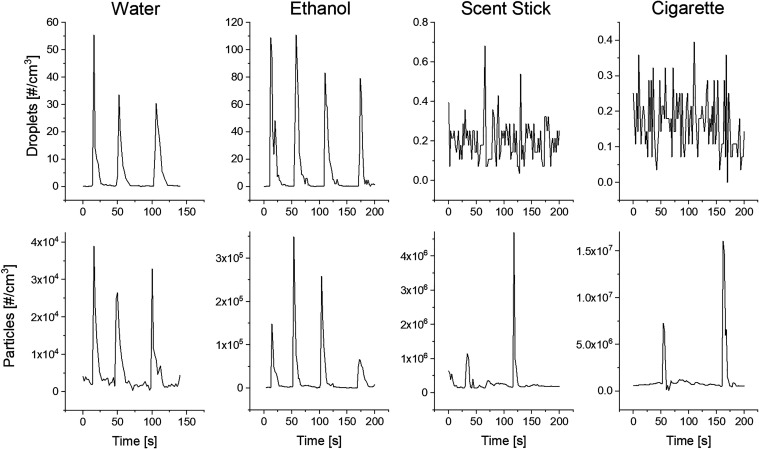
Proof of selectivity. Measured concentrations of droplets (top row) and particles (bottom row) during experiments involving sprayed water and ethanol, and emissions of particles from a smoldering incense stick and cigarette. Note that the scales differ due to the different concentration values recorded.

### Case Study

The first case study on droplet detection was conducted in a conference room (12 m × 6 m × 4 m), with 60 attendees present. The concentration of droplets, averaged over 20 s, was measured using the presented device. In parallel, an environmental sensor node (SEN55, Sensirion) was used to monitor PM_2.5_ and volatile organic compounds (VOCs). All the measurements were taken in the corner of the room near the lecturers’ area located next to one of the entrance doors. Air exchange was provided through four large windows along one of the longer walls of the room and through two entrance doors from the corridor.

The conference program consisted of three sessions (Figure 3, blue background), during which both the windows and the doors remained closed, and two breaks between sessions, during which attendees left the room, and both the windows and doors were opened for ventilation. At the beginning of the break following the second session, lunch was served in the corridor (Figure 3, hatched area). During the third session, the doors to the corridor were opened for 20 min (Figure 3, cross-hatched area).

**Figure 3:**
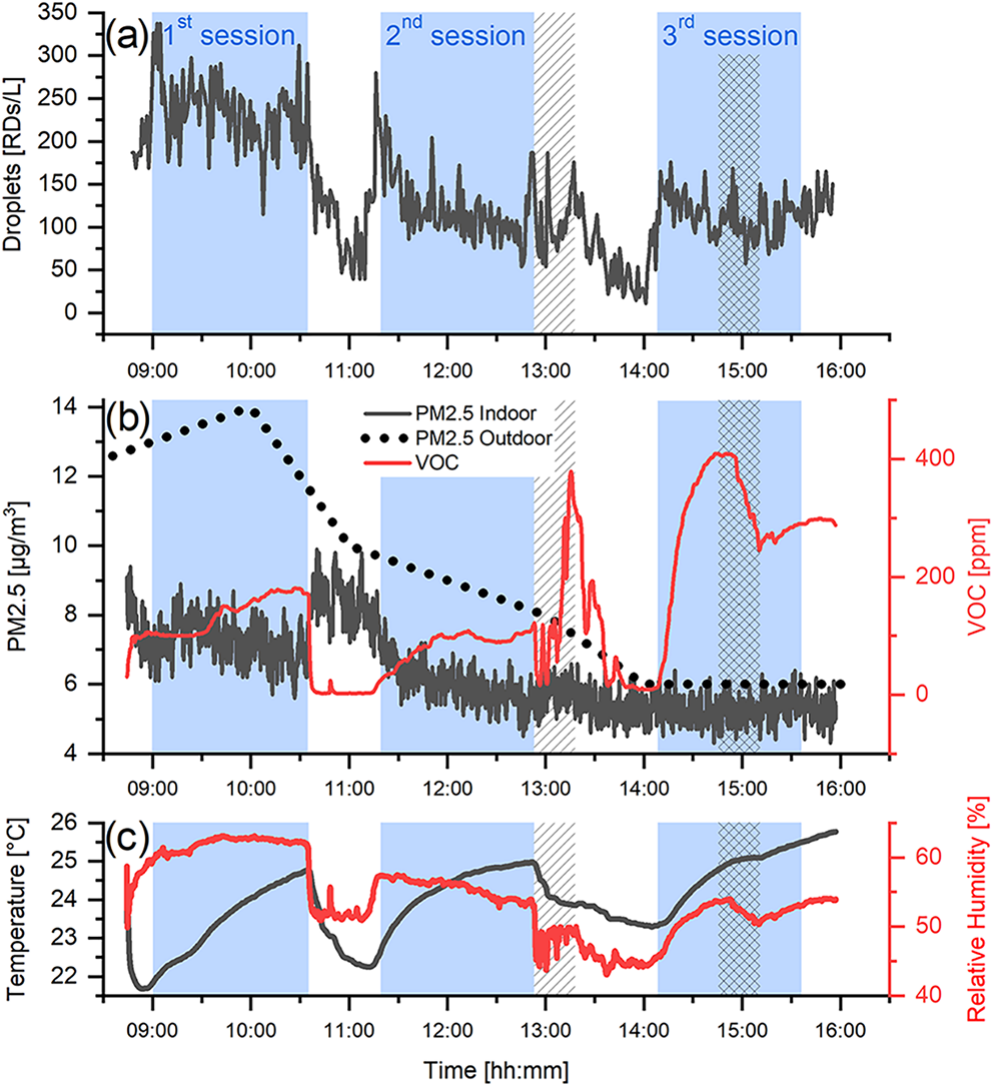
Time evolution of: (a) Respiratory droplet concentration; (b) indoor and outdoor PM_2.5_ and VOC levels; (c) temperature and relative humidity. The blue areas indicate time periods when the conference sessions were in progress, with attendees present and both the windows and doors closed. Prior to the first session and during the breaks, the windows were opened for ventilation. During the breaks, the participants temporarily left the room. The hatched area following the second session marks the lunch break, during which time the door to the corridor was open. The cross-hatched area during the third session indicates a short period when the doors to the corridor were briefly opened.

The droplet concentration in the conference room (Figure 3a), measured using the presented device, shows a clear decrease during the breaks when the attendees left the room, followed by an increase as soon as they returned. The average droplet concentration during the first session was 235 RDs/L, which dropped below 40 RDs/L by the end of the first break. Over the course of the day, the concentration of droplets decreased, reaching only 114 RDs/L during the third session. This reduction can be attributed to the decreasing number of attendees as the conference progressed. The number concentration of RDs at absence of human in the room at the end of lunch break (around 2 p.m.) was below 10 RDs/L.

The concentration of VOCs (Figure 3b) follows a similar trend to that of the droplets. It increased during the sessions when the attendees were present in the conference room and decreased when the windows or doors were opened. The rise in VOC levels can be attributed to the presence of people,^43^ which contribute up to 40% of the measured indoor VOC concentration.^44^ The observed correlation between the VOC and droplet concentrations shows that the detected droplets originated from the attendees.

In contrast, the indoor concentration of PM_2.5_ (Figure 3b) increased during the breaks when the windows were opened, most notably between the first and second sessions. The outdoor PM_2.5_ concentration^45^ peaked at 10 a.m. with 14 μg/m^3^ and then gradually decreased throughout the day, reaching 6 μg/m^3^ after 2 p.m. Since the outdoor PM_2.5_ concentration was higher than the indoor concentration during the break between the first and second sessions, it is likely that the observed increase in indoor PM_2.5_ was due to the infiltration of outdoor particles as a result of air exchange.

## Discussion

RDs are recognized as carriers of infectious pathogens and constitute a primary pathway for the transmission of airborne diseases.^2,3^ Exhaled droplets contain not only water but also salts, mucins (proteins), lung surfactant components, and pathogens.^46^ Larger respiratory droplets settle rapidly due to gravity, whereas smaller droplets remain airborne and are transported by surrounding airflows.^4,5^ Although these droplets begin drying immediately after exhalation, their composition never allows them to dry out completely,^6^ as evaporation is governed by multiple phenomena.^47^ First, evaporative cooling decreases the droplet surface temperature by approximately 10 K at 50% RH due to the high enthalpy of vaporization of water, which in turn reduces the evaporation rate.^47^ Second, since exhaled droplets contain not only water but also other constituents,^48^ which lower the chemical potential of water, thereby further reducing the evaporation rate.^47^ Third, as relative humidity decreases, homogeneously mixed organic–inorganic liquids in respiratory droplets can transition into amorphous, glassy, or gel-like states, in which diffusion and efflorescence are hindered, thereby inhibiting water evaporation.^49^ Furthermore, during rapid drying, the droplet core cannot equilibrate its water activity quickly enough,^50^ triggering a liquid–liquid phase separation of droplet components and the formation of a core–shell structure.^46^ The shell may consist of mucin,^46^ lipids,^7^ proteins,^7^ surfactants,^48^ or other nonvolatile fluids and acts as a physical barrier that encapsulates remaining water,^7^ significantly slowing evaporation.^47^

Exhaled droplets eventually reach an equilibrium state at which their size no longer changes.^7^ During this process, dried droplets shrink to 27–58% of their original radius.^47^ Under typical indoor relative humidities of 45–60%, droplets retain approximately 45–50% of their initial water content at equilibrium,^7^ and this retained moisture reduces the probability of viral inactivation by dehydration.^7^ These partially dehydrated, aerosolized droplets can remain suspended for prolonged periods, accumulate in indoor environments, and transport viruses and other pathogens that remain infectious for extended durations.^8^ Consequently, the detection of RDs is of critical importance for public health.

Various methods of RDs detection have been reported, but they are generally limited to the immediate vicinity of the exhalation source,^9,10,21–24^ rendering them unsuitable for the detection of RDs persisting in the indoor air. Techniques for the detection of airborne viruses within droplets are also available^17–20^ ; however, these approaches are typically restricted to specific, predefined viruses and require prolonged PCR procedures. Conventional particle counters have limitations for the detection of droplets.^1^ Due to the substantially higher concentration of solid aerosol particles compared to droplets, the specific signals associated with the droplets are hidden, leading to a lack of specific droplet-related information. In contrast, the method presented here enables the selective detection and quantification of droplets in indoor air, with the measurement data unaffected by the concentration of solid aerosol particles.

The developed device is based on the detection of change in capacitance when a droplet enters the sensor’s electric field. While capacitive sensing is commonly employed in humidity sensors, which can achieve subsecond response times,^51^ by utilizing a hygroscopic dielectric layer (e.g., polyimide)^52,53^ to absorb water vapor, consequently increasing its permittivity and absolute capacitance,^54^ our device is fundamentally different. Our sensor employs a dielectric with an extremely low water absorption coefficient (0.10 wt %; ASTM D570), a value approximately 50 times lower than that of typical polyamides,^55^ rendering the device inherently insensitive to ambient RH. Furthermore, electronic circuitry is specifically designed to detect rapid capacitance changes (in the microsecond range) rather than measuring an absolute capacitance. The design choice prevents the detection of significantly slower capacitance changes such as those caused by RH fluctuations. This selective response has been experimentally confirmed, demonstrating that the detector’s signal is uncorrelated with RH fluctuations and is exclusively responsive to airborne droplets (SI 1). It should be noted that the signal amplitude can be influenced by ambient humidity, primarily through its effect on droplet evaporation and growth. While phenomena such as droplet nucleation at RH saturation could also influence the signal, these conditions are rarely encountered in typical indoor environments.

The device can detect individual droplets, with a time resolution of approximately 10 μs, enabling the detection of up to 10^5^ droplets/s. With an airflow rate of 1 L/min employed in the device, concentrations of up to 6000 droplets/cm^3^ can be measured. It can measure droplets with diameters greater than 200 nm ± 100 nm (SI 2). However, the size distribution of the droplets cannot be directly determined from the signal’s amplitudes, as the measured capacitance change also depends on the location of a droplet’s impact on the sensor surface due to spatial variations in the electric field strength. The specific coaxial geometry of the presented coplanar capacitor necessitates numerical simulations,^56^ which are planned for future work. The present detection method only requires droplets to pass through the electric field between the electrodes. This principle enables the use of diverse electrode geometries, such as those with parallel or tangential droplet trajectories relative to the electrodes, or a channel design with electrodes on opposing sides.

In addition to the static description, highly accelerated and then suddenly halted droplets also require a dynamic insight. According to theoretical calculations, the impact of a liquid droplet against a solid surface at a high impact velocity can lead to the formation of traveling pressure-propagation fronts within the droplet, propagating in a direction perpendicular to the surface.^57^ The liquid is compressed and the intermolecular interaction between the locally increased number of molecules is strengthened, thereby improving the dielectric response.^58^ The estimated contact pressure exposed to the sensor surface by the impact of water droplets with a velocity of 130 m/s is around 100 MPa. The contribution to the dielectric constant of water due to this pressure is around 1%. Another contribution to the signal can be related to triboelectric charges on the surfaces of the droplets, which were exposed to the friction with air/nozzle or with other air pollutants. These charges are positive or negative depending on the counterpart the water drop has interacted with.^59^ When the charged water droplets hit the dielectric surface, they affect the electric field of the sensor according to their polarity.

The case study, conducted in a conference room with approximately 60 attendees, demonstrated that RD concentrations can be selectively measured in real time. Indoor air may contain water droplets originating from sources such as humidifiers, showers, toilets, cooling and cleaning sprays, misting HVAC systems, and plant transpiration. These droplets can compromise measurement accuracy if the sensor is placed too close to their origin. However, unlike respiratory droplets, such water droplets typically evaporate rapidly under unsaturated RH conditions. At normal indoor RH levels (40–60%), pure water droplets are not present in the air unless they are intentionally introduced. To minimize potential interference, the sensor location should be carefully selected, either by maintaining sufficient distance from these sources or by orienting the sensor to avoid direct exposure to the droplet flow. In the present case study, measurements were conducted in a conference room where none of the above-mentioned sources of non-respiratory water droplets were present, ensuring that there were no interfering droplets present. It is therefore reasonably to conclude that the detected droplets were primarily of human origin. The measured concentration ranged from 40 RDs/L (0.04 RDs/cm^3^) when the room was unoccupied and ventilated through open windows, to 330 RDs/L (0.33 RDs/cm^3^) when no ventilation was performed. The presence of people in a nonventilated room with a volume of around 300 m^3^ increased the average number concentration of RDs by several times in 90 min.

The related studies reported the concentrations of RDs produced during specific activities: breathing (0.1 RDs/cm^3^),^60^ speaking (0.004–0.223 RDs/cm^3^),^21^ sustained vocalization (1.1 RDs/cm^3^),^60^ and for coughing (2.4–5.2 RDs/cm^3^).^21^ However, these measurements were performed in close proximity to a human mouth and did not account for the subsequent dispersion of droplets within indoor spaces, where various physical processes affect their behavior. Large droplets tend to settle rapidly due to gravitational forces, whereas smaller droplets are subject to thermodynamic processes, such as evaporation, collisions, charging, secondary nucleation, and translocation by ambient airflow. Furthermore, following exhalation, the volume of the exhaled puff expands through mixing and dilution with the surrounding air, leading to a decrease in the droplet concentration.^1,61^ Aerosolized droplets can accumulate and remain infectious in indoor air for several hours.^62^ As a result of these processes, the concentration of RDs in the close vicinity of individuals who produced them can differ from the concentration of RDs in enclosed indoor environments.

The presented method does not reveal whether the detected RDs contain viruses or other pathogens. Previous research indicates that the average viral RNA load for COVID-19 is approximately 7 × 10^6^ virions per milliliter.^63^ Based on this value, there is a 0.37% probability that a 10-μm droplet contains at least one virion.^64^ Since around 600 virions are estimated to be required to cause an infection with the original COVID-19 strain,^65^ we can approximate the infection risk. Assuming an average breathing rate of 6 L/min at rest^66^ and a RDs concentration of 100 RDs/L, it would take roughly 37 min of continuous indoor breathing to inhale enough droplets for a potential infection. However, if the virions act synergistically rather than individually, the required infectious dose might be lower.^67^ Furthermore, infection rates vary significantly based on age and gender, with some individuals being infected much faster than the 37 min average time.^68^

In current practice, indoor-air safety is predominantly maintained through continuous mechanical ventilation. It has been demonstrated that increasing the air exchange rate to 10 air changes/h (ACH) can reduce the infection risk by approximately one-third when compared to poorly ventilated environments.^69^ Moreover, the Centre for Disease Control and Prevention recommends a minimum of 12 ACH for airborne-infection isolation rooms.^70^ However, these ventilation strategies are typically implemented without real-time feedback on the actual concentration of RDs, the primary carriers of airborne pathogens, in indoor air. While CO_2_ levels can serve as a proxy for ventilation control, relying solely on absolute CO_2_ concentration thresholds is inadequate for assessing the airborne transmission risk, as CO_2_ does not directly correlate with the dynamics of pathogen-laden aerosols.^71^ Furthermore, PM_2.5_ measurements also cannot serve as reliable indicator of indoor air quality in the context of aerosolized pathogen transmission, because outdoor PM_2.5_ and human activity can contribute to elevated levels, and because conventional PM_2.5_ sensors lack selectivity for detecting RDs.

The selective, real-time detection capability of the device presented in this study offers a major advance in airborne-transmission control. By providing continuous and direct measurements of RD concentrations, the presented device enables dynamic ventilation strategies that are responsive to real-time indoor-air conditions. This could significantly reduce the energy and economic burden of overventilation, while enhancing infection-control measures. Furthermore, the ability to monitor the droplet distribution could help to establish new standards for air quality and pathogen-exposure risk in shared spaces. Knowledge of low RD concentrations in indoor air could serve as an indicator of a safer environment regarding respiratory-infection transmission. As such, the developed technology has the potential to transform current indoor-air safety paradigms and public-health management.

## Conclusion

This study presents the first method and device for the selective detection of individual RDs in indoor air. The detection principle is based on the difference in dielectric constants between droplets and solid aerosol particles, enabling reliable discrimination. The device is capable of the real-time detection of up to 6,000 droplets/cm^3^ with diameters greater than 200 nm ± 100 nm. The case study demonstrated that the droplet concentration in an empty, ventilated room was approximately 40 RDs/L, while a 60-person occupancy raised it to 330 RDs/L. Furthermore, the results indicate that the RD concentration is not correlated with the PM concentration. Given that the concentration of RDs is at least 4 orders of magnitude lower than that of nanoparticles, their presence is typically hidden in conventional particle measurements. This highlights the need for a selective method to accurately monitor droplets that may carry infectious viruses and other pathogens and contribute to respiratory disease transmission. In addition to monitoring human or animal RDs, the detection method is also suitable for identifying water-containing particles, such as pollen or bioaerosols. Moreover, the device can be used to determine the dielectric constant of submicron particles with narrow size distributions, broadening its applicability in aerosol research and public-health monitoring.

## Supplementary Material



## Data Availability

The data that support the findings of this study are available within the article, its Supporting Information file, and in Zenodo, https://doi.org/10.5281/zenodo.15489441.

## Notes

Experimental results demonstrating the detector’s response to water droplets and insensitivity to changes in humidity; experimental results determining the smallest detectable droplet size (PDF)

## Abbreviations

ACH: air changes per hour
CPC: condensation particles counter
OPC: optical particle counter
PCB: printed-circuit board
PM: particulate matter
RH: relative humidity
RD: respiratory droplet
RSV: respiratory syncytial viruses
RT-qPCR: reverse transcription-quantitative polymerase chain reaction
VOC: volatile organic compounds
