# Supplementary material for: The Selective Detection of Individual Respiratory Droplets in Air
Source: ACS Sens. 2025 Dec 16;11(1):55–62. doi: 10.1021/acssensors.5c02057 (PMC12836340; doi:10.1021/acssensors.5c02057)
Supplement: Supplementary file 1 [file se5c02057_si_001.pdf]

SUPPLEMENTARY INFORMATION FOR

The selective detection of individual respiratory droplets in air

Matjaž Malok[1,2], Darko Kavšek[1], Maja Remškar [1,3]***

[1]Jozef Stefan Institute, Ljubljana, Slovenia

[2]Faculty of Mathematics and Physics, University of Ljubljana, Ljubljana, Slovenia

[3]Nanotul Ltd., Ljubljana, Slovenia

*E-mail: matjaz.malok@ijs.si

**E-mail: maja.remskar@ijs.si

SI 1: Droplet detector response regardless of humidity changes

To demonstrate the detector's response for droplets and not to water vapour (humidity), a controlled experiment was performed within a tightly closed chamber (0.75 × 0.6 × 0.6 m). The relative humidity (RH) in the chamber was monitored using a Sensirion SEN55x sensor node.

The experimental procedure was as follows: Water vapor was introduced into the chamber by inductive heating of water, causing a rapid increase in RH inside the chamber. Once boiling commenced, the heating was switched off. After several minutes, the chamber door was opened, allowing the RH to gradually decrease to an ambient level.

The results clearly differentiate the detector's response from the humidity signal (**Figure SI 1**). The SEN55x sensor recorded a sharp increase in RH during the initial boiling phase. In contrast, the droplet detector showed no significant increase in signal, remaining at its baseline level (attributed to background droplets level). After boiling ceased, the water remained hot and continued to evaporate. When the RH approached 94%, the droplet detector's signal exhibited a distinct peak, indicating that at this RH, the nucleated droplets did not dry out and persisted in the air as detectable liquid particles. When evaporation decreased, the concentration of detected droplets returned to a level similar to that at the beginning of the experiment.

When the chamber door was opened, the RH began to decrease. Concurrently, the droplet detector recorded a slight increase in droplet concentration, corresponding to the influx of external droplets from the surrounding environment.

This experiment provides direct evidence that the detector's signal is uncorrelated with ambient humidity fluctuations and is exclusively responsive to the presence of droplets in the air.

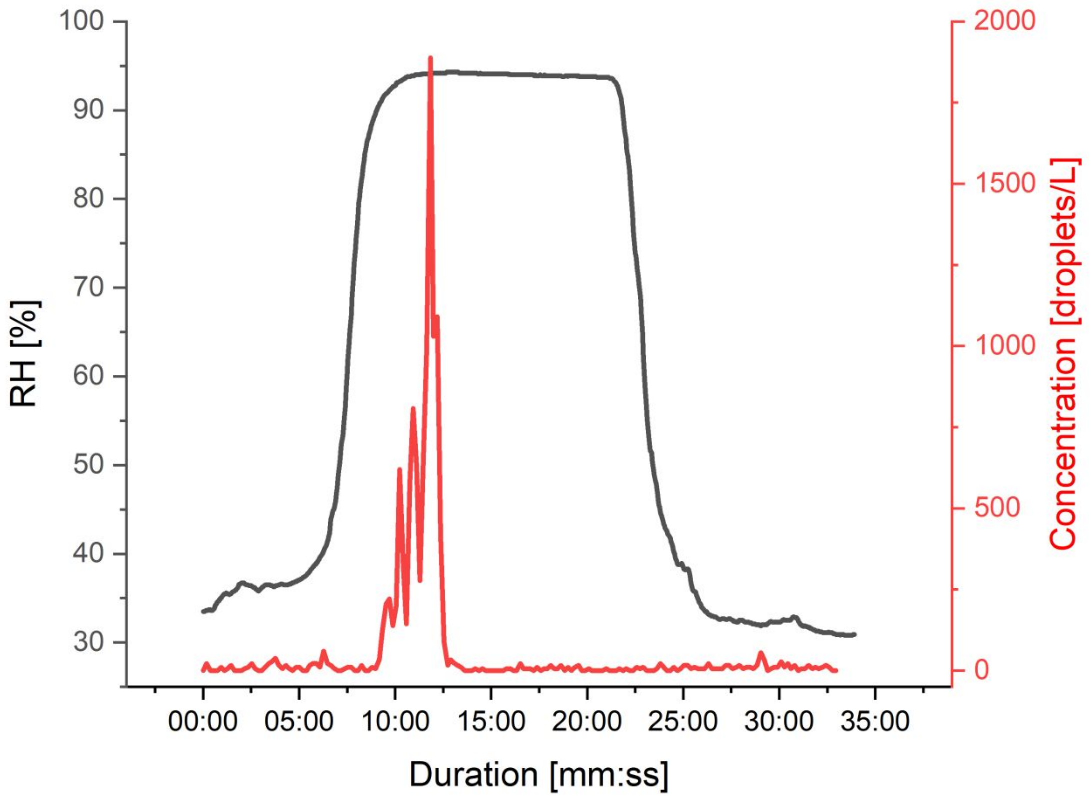

Figure SI 1: Uncorrelated response of droplet concentration and relative humidity. Relative humidity (black line) and droplet concentration (red line) were measured simultaneously. The detector's signal is specific to droplets and is unaffected by fluctuations in relative humidity.

SI 2: Calibration of the droplet detector

The size of the smallest detectable droplet was estimated experimentally by counting aerosolized TiO_2 particles (Sigma Aldrich, CAS 1317-80-2, No. 224227, rutile, 99.9%; <5 micron; <10 % of anatase). For the calibration, a dielectric constant of 23 was used, corresponding to a mixture of rutile and anatase phases.[SI_1]

TiO_2 particles were first size-separated by sedimentation of their dispersion in isopropanol. After 20 s of the sedimentation, the supernatant was transferred to a glass jar and dried at 80 °C for 12h. The SEM image of these particles is shown in **Figure SI 2**a. The particle size, automatically determined by computer vision, ranged from 250 nm to 900 nm with an average size of around 575 nm (**Figure SI 2**a, c). A background measurement representing the normal size distribution of droplets in indoor air was first recorded (**Figure SI 2**, red line). Subsequently, a glass jar equipped with inlet and outlet tubes was connected to the detector inlet, and air was drawn through the jar containing TiO_2 particles dried on its bottom. The particles were aerosolized by applying a pressure drop - achieved by compressing the tubes, allowing a vacuum to form, and then releasing them - and transported into the detector. A high particle concentration was observed, reaching up to 1.7 particles/cm^3. Most signals exhibited low amplitudes, consistent with small particle sizes, suggesting that the majority of detected particles were not agglomerated. The average size of particles collected on the sensor surface was approximately 580 nm, with no particles smaller than 380 nm observed (**Figure SI 2**b, c).

Considering the dielectric constant of water (ε_w) of 78.2,[SI_2] and TiO_2 (ε_p) of 23,[SI_1] the typical size range of most TiO_2 particles between 300 and 900 nm (**Figure SI 2**c), and the dependence of capacitance change on droplet or particle volume, the diameter of the smallest detectable water droplets (D_w) is estimated to be approximately 100 nm. This estimate is based on the

equation: $D_w = (\varepsilon_P/\varepsilon_w)^{1/3}.D_{p}$., where D_p is diameter of the aerosolized TiO_2 particles. Since it is not possible to ascertain whether the smallest TiO_2 particles observed on the sensor surface were responsible for the smallest measurable signal, an additional estimation was performed using TiO_2 particles with a diameter of 900 nm - representing the upper bound of the predominant particle size distribution (**Figure SI 2**c). Under this assumption, the corresponding water droplet diameter would be approximately 300 nm. Based on these considerations, the lower detection limit for droplet size is estimated to lie between 100 nm and 300 nm. For larger droplets, the only limiting factor is the nozzle diameter, which is 400 µm. Consequently, the droplet detection system is capable of detecting droplets with diameters ranging from 200 nm ± 100 nm up to 400 µm.

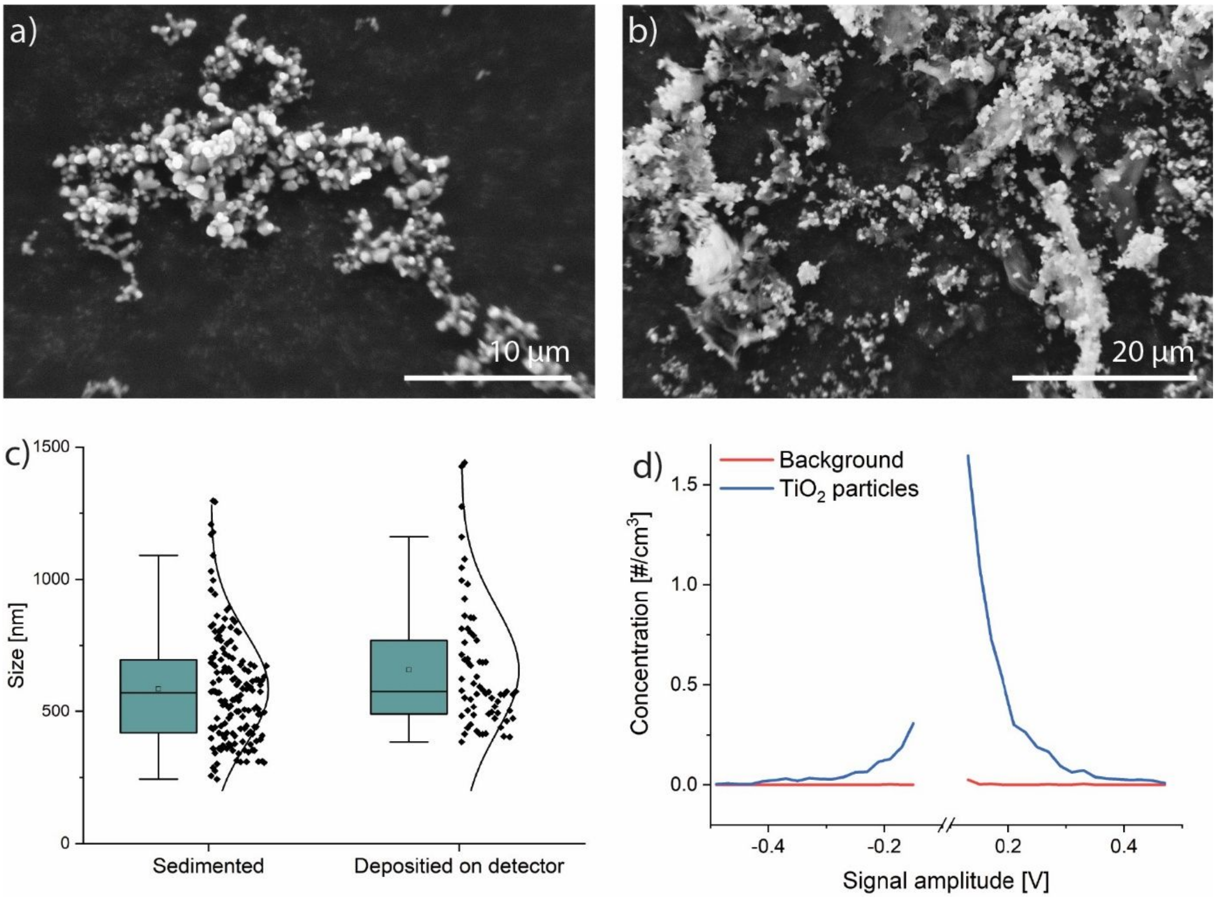

Figure SI 2: TiO_2 particles. a) SEM image of particles dried from the supernatant at the bottom of a glass jar; b) SEM image of particles deposited onto the sensor surface; c) Size distribution of particles shown in (a) and (b). d) Measured concentration of particles as a function of signal amplitude (red: background; blue: TiO_2 particles aerosolized under air pressure drop).

References:

(SI_1) Wypych, A. et al. Dielectric Properties and Characterisation of Titanium Dioxide Obtained by Different Chemistry Methods. *J. Nanomater*. **1**, 124814 (2014).

(SI_2) Britannica, The Editors of Encyclopaedia Britannica. "Dielectric constant". Encyclopedia Britannica, https://www.britannica.com/science/dielectric-constant (accessed 1 Feb 2025).
